# Competition between Usutu virus and West Nile virus during simultaneous and sequential infection of *Culex pipiens* mosquitoes

**DOI:** 10.1080/22221751.2020.1854623

**Published:** 2020-12-14

**Authors:** Haidong Wang, Sandra R. Abbo, Tessa M. Visser, Marcel Westenberg, Corinne Geertsema, Jelke J. Fros, Constantianus J. M. Koenraadt, Gorben P. Pijlman

**Affiliations:** a Laboratory of Virology, Wageningen University & Research, Wageningen, Netherlands; b Laboratory of Entomology, Wageningen University & Research, Wageningen, Netherlands; c Dutch National Plant Protection Organization (NPPO-NL), Wageningen, Netherlands

**Keywords:** Usutu virus, West Nile virus, competition, mosquito, vector competence

## Abstract

Usutu virus (USUV) and West Nile virus (WNV) are closely related mosquito-borne flaviviruses that are mainly transmitted between bird hosts by vector mosquitoes. Infections in humans are incidental but can cause severe disease. USUV is endemic in large parts of Europe, while WNV mainly circulates in Southern Europe. In recent years, WNV is also frequently detected in Northern Europe, thereby expanding the area where both viruses co-circulate. However, it remains unclear how USUV may affect the future spread of WNV and the likelihood of human co-infection. Here we investigated whether co-infections with both viruses in cell lines and their primary mosquito vector, *Culex pipiens*, affect virus replication and transmission dynamics. We show that USUV is outcompeted by WNV in mammalian, avian and mosquito cells during co-infection. Mosquitoes that were exposed to both viruses simultaneously via infectious blood meal displayed significantly reduced USUV transmission compared to mosquitoes that were only exposed to USUV (from 15% to 3%), while the infection and transmission of WNV was unaffected. In contrast, when mosquitoes were pre-infected with USUV via infectious blood meal, WNV transmission was significantly reduced (from 44% to 17%). Injection experiments established the involvement of the midgut in the observed USUV-mediated WNV inhibition. The competition between USUV and WNV during co-infection clearly indicates that the chance of concurrent USUV and WNV transmission via a single mosquito bite is low. The competitive relation between USUV and WNV may impact virus transmission dynamics in the field and affect the epidemiology of WNV in Europe.

## Introduction

Pathogenic arthropod-borne (arbo)viruses are transmitted by blood-feeding vectors e.g. mosquitoes, ticks, midges or sand flies. Arboviruses that are transmitted by the same vector species are more likely to co-circulate, making arbovirus co-infection a potential public health concern [1]. In Latin America, human co-infections with Zika, dengue, chikungunya or other arboviruses are increasingly reported [2–4], and this complicates accurate diagnosis of patients. The primary vector for these viruses, the *Aedes aegypti* mosquito, can become infected with more than one virus at a time and subsequently co-transmit these viruses to the next human host [5,6]. In Europe, two closely related arboviruses, Usutu virus (USUV) and West Nile virus (WNV) (family *Flaviviridae*, genus *Flavivirus*) have been co-circulating for more than two decades [7]. Both viruses are mainly transmitted between avian species by the common house mosquito *Culex pipiens* [7,8] and can cause severe disease in humans. However, it remains unknown whether *Cx. pipiens* is able to co-transmit both viruses and whether circulation of one virus affects the transmission dynamics of the other.

In the summer of 2016, a major USUV outbreak was reported in Belgium, France and the Netherlands, resulting in mass mortality of blackbirds and captive owls [9–11]. This indicates that this African-origin flavivirus has substantially expanded its territory since it first emerged in Southern Europe in 1996 [12]. Although human USUV infections are sporadically reported and often remain asymptomatic, recent clinical data has revealed an association of USUV infections with neurological disorders in both immunocompromised and immunocompetent patients [13–18]. USUV pathogenicity in humans is not well understood and requires more scientific attention. Pioneering studies in mouse models have indicated that USUV can invade and replicate in the murine nerve system and cause strong inflammation in the spinal cord and brain, which is in line with the neurological disorders in humans that were attributed to USUV infection [19,20].

In addition to USUV, WNV has been circulating in Southern Europe for decades [21]. The enzootic transmission cycle of WNV is very similar to that of USUV. Both viruses are efficiently transmitted by *Cx. pipiens* mosquitoes, mostly between *Passeriformes* birds, with the Eurasian blackbird (*Turdus merula*) and carrion crow (*Corvus corona*) displaying a high susceptibility for both viruses [22]. Similar to USUV, humans and other mammals are incidentally infected by WNV, however the outcome of a WNV infection in these species is more frequently associated with febrile illness, neurological disease and even death [23,24].

**Figure 1.**
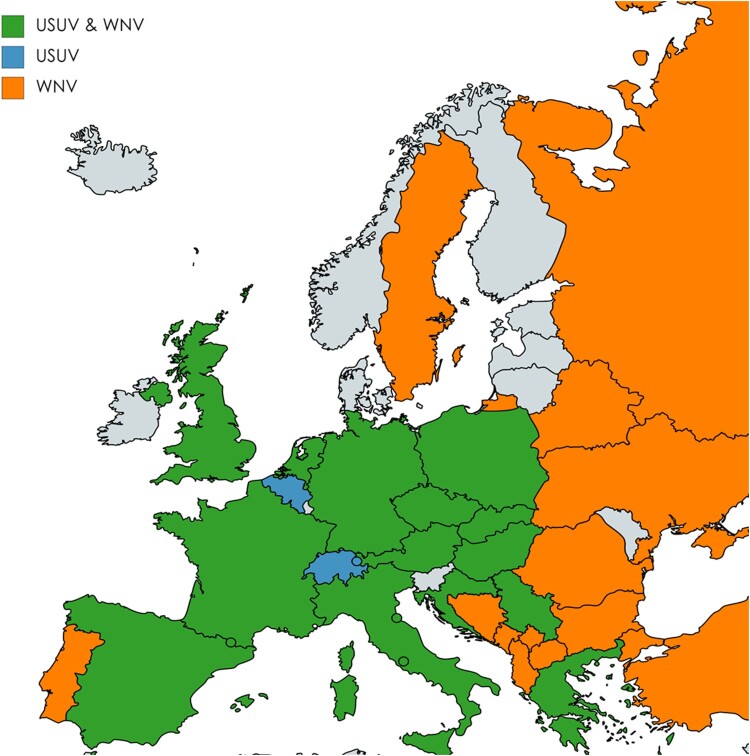
Co-circulation of Usutu virus (USUV) and West Nile virus (WNV) in European countries. The map shows European countries where USUV, WNV or co-infection cases are reported according to the data provided by European Centre for Disease prevention and control (ECDC) and literatures. Countries where both USUV and WNV co-circulate are represented in green. Countries where only USUV or WNV circulates are represented in blue or orange, respectively. The map was generated by using the free online tools https://mapchart.net/europe.html.

Despite the wide spread of *Cx pipiens* vectors and reservoir bird hosts across Europe, WNV dispersal is mainly reported in Southern Europe [25,26]. However, increasing activity of WNV has recently been recorded in more Northern European regions, which has enlarged the geographic overlap between both viruses (Figure 1) [7,27–31]. In 2018, the first ever human USUV and WNV co-infection case was identified among blood donors in Austria [30,32]. It remains unclear whether this is due to a single bite by a co-infected mosquito or sequential bites by USUV and WNV infected mosquitoes. Considering the large geographic overlap of both viruses and the northwards spreading trend of WNV, it is important to understand to what extent co-circulation of USUV and WNV may affect the transmission dynamics of both viruses. In the current study, virus replication during co-infection of both USUV and WNV was studied in mammalian, avian and mosquito cells. Furthermore, the effect of simultaneous and sequential USUV-WNV infection on the transmissibility of both viruses by *Cx. pipiens* mosquitoes was investigated.

## Materials and methods

### Cells and viruses

African green monkey kidney Vero E6 cells and chicken embryo DF-1 cells were maintained in Dulbecco's Modified Eagle Medium (DMEM, Gibco, Carlsbad, CA, USA) supplemented with 10% foetal bovine serum (FBS, Gibco), penicillin (100U/ml, Sigma-Aldrich, Saint Louis, MO, USA) and streptomycin (100 µg/ml, Sigma-Aldrich) at 37°C with 5% CO_2_. *Culex tarsalis* Cx.t cells were grown in Schneider's *Drosophila* medium (Gibco) supplemented with 10% FBS at 28°C. *Aedes albopictus* C6/36 cells were grown in Leibovitz L-15 medium (Gibco) supplemented with 10% FBS, 2% tryptose phosphate broth (Gibco) and 1% nonessential amino acids (Gibco) at 28°C. Passage 5 and 6 USUV stock, the Netherland 2016, black bird isolate (lineage Africa 3, GenBank accession no. MH891847.1) and passage 2 WNV stock Greece 2010 (lineage 2, GenBank accession no. HQ537483.1) were grown and titrated on Vero E6 cells.

### Virus infection

For growth curve analysis, USUV P5 or WNV P2 were inoculated to Vero, DF-1, C6/36 and Cx.t cells at multiplicity of infection (MOI) of 0.1. Supernatants were harvested at 0, 1, 2, 3, 4 and 7 days post infection (dpi) and titrated by end point dilution assay (EPDA) on Vero cells. For co-infection, USUV P5 and WNV P2 stocks were inoculated to each cell type simultaneously at different MOI combinations: a (USUV = 0.1, WNV = 0), b (USUV = 0.1, WNV = 0.1), c (USUV = 0.1, WNV = 5), d (USUV = 5, WNV = 0), e (USUV = 5, WNV = 0.1) and f (USUV = 5, WNV = 5). At 3 dpi, culture medium was removed, and cells were lysed by TRIzol reagent (Invitrogen). RNA of the cell lysates was extracted and used for subsequent analysis by TaqMan qPCR.

### Simultaneous infection of *Culex pipiens* mosquitoes

The *Cx. pipiens* (biotype *pipiens*) laboratory colony, originating from Best, the Netherlands was established in 2016 and maintained at 23°C with 16:8 (L:D) photocycle and 60% relative humidity (RH) [33]. Freshly obtained chicken blood containing 5×10^6^ TCID_50_/ml USUV P5 and 5×10^6^ TCID_50_/ml WNV P2 was fed to *Cx. pipiens* mosquitoes (∼7 days old) using a Hemotek feeder system. Infectious blood meal containing either USUV or WNV at 5×10^6^ TCID_50_/ml was offered as control. The fully engorged females were selected and maintained at 28°C with 16:8 (L:D) photoperiod. Three engorged mosquitoes were stored at −80°C right after the selection to determine the amount of virus ingested by the mosquitoes (Figure S1).

### Sequential infection of *Cx. pipiens* mosquitoes

*Cx. pipiens* were first exposed to either a virus-free or an infectious blood meal containing 5×10^6^ TCID_50_/ml USUV P6 stock. The fully engorged mosquitoes were subjected for a second infectious blood meal containing 5×10^6^ TCID_50_/ml of WNV P2 7 days later. Oviposition cups were provided between the subsequent blood meals. To bypass the mosquito midgut, 69nl of USUV P6 stock (approximately 2400 TCID_50_) or the same volume of DMEM medium was injected into the mosquitoes intrathoracically using a Drummond Nanoject II injector (Drummond Scientific, Broomall, PA, USA). The injected mosquitoes were kept for 7 days before orally exposed to an infectious blood meal containing 5×10^6^ TCID_50_/ml of WNV P2 stock.

### Salivation and infectivity assay

Mosquito saliva and body homogenate were collected according to the previously reported forced salivation technique [25]. Saliva and the supernatant of body homogenate were inoculated to Vero cells. Cytopathic effects (CPE) was scored on day 3 and 6 post infection. CPE positive cells as well as the corresponding mosquito samples were subjected to RNA extraction and TaqMan qPCR (see below). Mosquito samples were determined as co-infection when both USUV and WNV were observed in either the Vero cells (that showed CPE) or the corresponding mosquito samples by TaqMan qPCR.

### RNA extraction

TRIzol reagent (Invitrogen) was used for total RNA isolations from cells and mosquito body homogenates according to the manufacture's instruction. Mag-Bind Viral RNA 96 kit (Omega) was used to isolate RNA from mosquito saliva samples. The yields of RNA samples were determined by Nanodrop (Thermo).

### Duplex TaqMan qPCR

To simultaneously detect USUV and WNV viral RNA, a duplex TaqMan qPCR system was developed. Primers and probes targeting the non-structure gene 5 (NS5) for both USUV and WNV were newly designed (Table S1) and synthesized (Integrated DNA Technologies, IDT). T7 RNA standards of USUV and WNV were generated based on a ∼900 bp PCR amplicon. The *in vitro* transcript viral RNA was quantified by Nanodrop and used to make a 10-time dilution series. Viral genome copies were calculated by online tool (http://endmemo.com/bio/dnacopynum.php). The TaqMan qPCR reaction was performed in a CFX96 Real-Time PCR instrument (Bio-Rad) with a 20 µl reaction system using the TaqMan^TM^ RNA-to-C**_T_**^TM^ 1 step Kit (Applied Biosystems). The amplification efficiency (AE) for both primers and probe sets were comparable between single and duplex assays (Figure S2). Positive USUV and WNV samples were determined by introducing a conservative cut-off Ct value of 34 based on a specificity test (Table S2). The cut-off value roughly corresponds to approximately 110 copies of USUV RNA and 250 copies of WNV RNA (Table S2 and Figure S3).

### Statistics

Fisher's exact test was used to compare the infection and transmission rate between different treatments. Student's t-test was used to compare the means of viral genome copies between two groups. One-way ANOVA with Tukey's multiple comparison or Kruskal–Wallis with Dunn's multiple comparison was used to compare the viral genome copies among more than two data sets. To explore the relation between viral genome copies and MOI combination during co-infection, an ANOVA model (Viral genome copies = MOI_USUV + MOI_WNV + cell + MOI_USUV * MOI_WNV) was performed. Statistics were done in R environment with default built-in package [34] and Prism v5.

## Results

### USUV is outcompeted by WNV in cells

**Figure 2.**
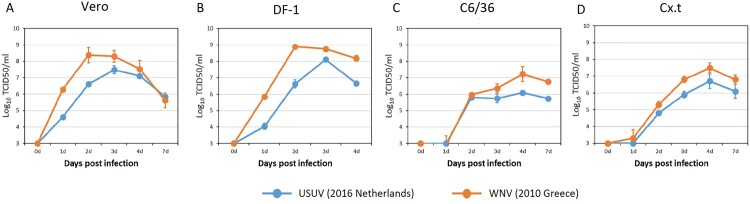
Growth kinetics of USUV (the Netherlands 2016) and WNV (lin2 Greece 2010). (A) Vero (green monkey), (B) DF-1 (chicken) and (C) C6/36 (*Aedes albopictus* mosquito) and (D) Cx.t (*Culex tarsalis* mosquito) cells were infected with either USUV or WNV at a multiplicity of infection (MOI) of 0.1. Virus titers were determined by end point dilution assay on Vero cells. Error bars represent the standard deviation (SD).

Prior to co-infection experiments we determined the replication kinetics of contemporary strains of USUV (lineage Africa 3, the Netherlands 2016) and WNV (lineage 2, Greece 2010) individually. One-step growth curves showed that WNV replicated faster and to higher titers than USUV in Vero, DF-1, C6/36 and Cx.t cells (Figure 2).

**Figure 3.**
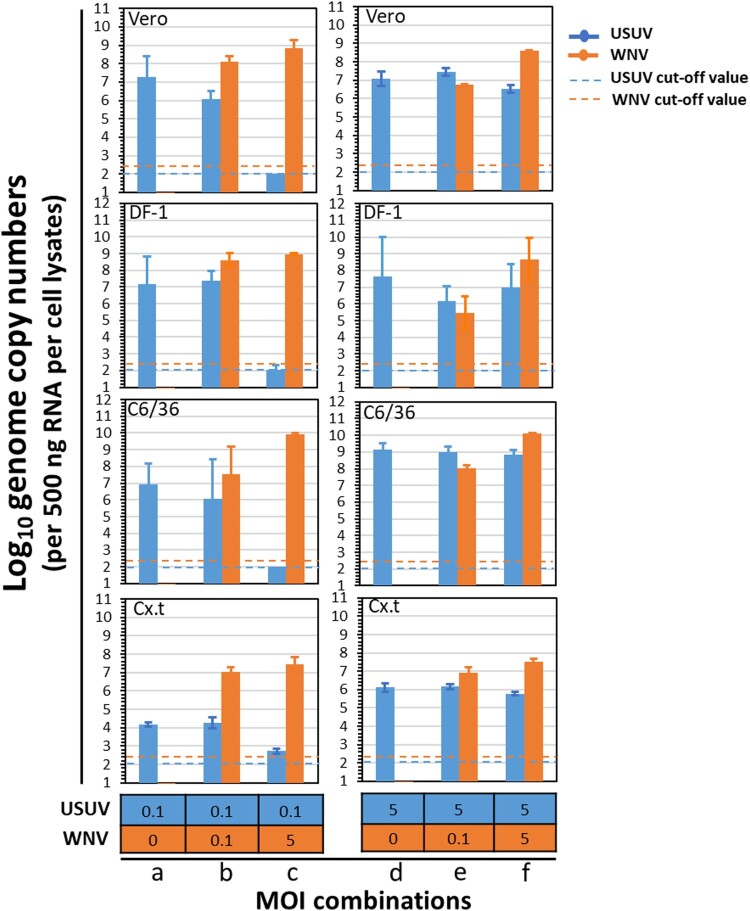
Co-infection of USUV (the Netherlands 2016) and WNV (Greece 2010) in cells of different origin. Vero, DF-1, C6/36 and Cx.t cells were co-infected with both USUV and WNV at multiplicity of infection of either 0, 0.1 or 5. At 3 days post infection (dpi), total RNA of the cells was extracted using TRIzol reagent. Five hundred nanogram of total RNA per sample was used for TaqMan qPCR. Error bars represent the standard deviation (SD). The blue and orange dash lines represent the cut-off value for USUV and WNV at Ct value of 34, respectively.

To investigate the putative interaction between USUV and WNV during co-infection, cells were simultaneously infected with both viruses at different MOI combinations. In cells that were infected with USUV only, at either a MOI of 0.1 or 5, USUV genome copies were all above 10^6^ (per 500 ng RNA) (Figure 3, MOI combinations a and d), except in Cx.t cell where the viral genome copies were around 10^4^ (per 500 ng RNA). When cells were simultaneously infected with a low amount of USUV (MOI = 0.1) and a high amount of WNV (MOI = 5), USUV genome copies were either below or close to the detection limits, whereas WNV genome copies were higher than 10^7^ (per 500 ng RNA) (Figure 3, MOI combination c). When cells were co-infected with both viruses at a same MOI of either 0.1 or 5 (Figure 3**,** MOI combinations b and f), USUV viral genome copies were either comparable or lower to that of the single USUV infection (Figure 3, MOI combination a and d). When a high MOI of USUV (5) and low MOI of WNV (0.1) were applied (Figure 3, MOI combination e), WNV genome copies were also at a similar level to that of USUV and USUV replication was also able to reach a comparable level to the single infection (Figure 3, MOI combination d) with viral genome copies ranging from 10^6^ to 10^9^ (per 500 ng RNA). We also observed that the WNV genome copies are only significantly related to the WNV MOI (ANOVA, F = 27.10, df = 1, *p* < 1×10^−8^) whereas the USUV genome copies were significantly related to USUV MOI (ANOVA, F = 58.48, df = 1, *p* < 1×10^−8^), WNV MOI (ANOVA, F = 41.50, df = 1, *p* < 1×10^−6^) and the interaction between USUV and WNV MOI (ANOVA, F = 30.11, df = 1, *p* < 1×10^−5^) (Table S3). This indicates that USUV experiences clear competition from WNV during co-infection. Together, the *in vitro* co-infection experiments showed that USUV is outcompeted by WNV in mammalian, avian and mosquito cells.

### USUV is outcompeted by WNV in *Culex pipiens* mosquitoes

**Figure 4.**
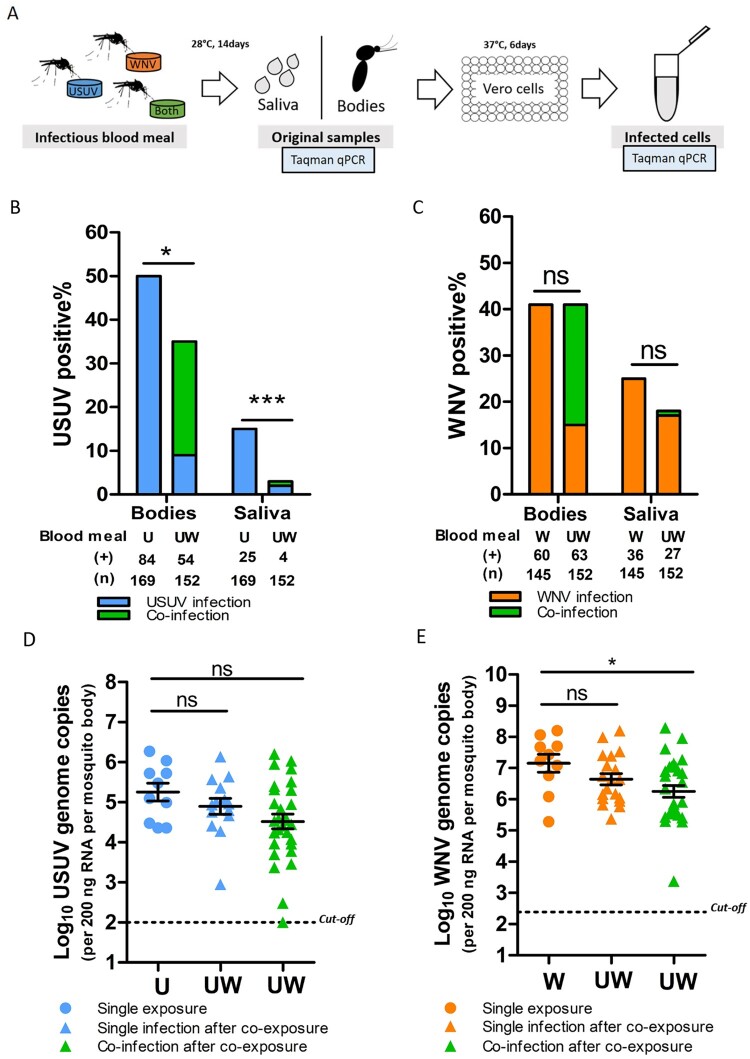
*Culex pipiens* selectively transmit WNV when exposed to both viruses through blood meal. (A) Schematic overview of the experimental design. (B, C) Bar graphs show the percent of USUV and WNV positive mosquito bodies and saliva of the total tested engorged mosquitoes at 14 days after blood meal. “**U**”, “**W**” and “**UW**” represent USUV, WNV and co-infectious blood meal, respectively. (**+**) and (**n**) indicate the numbers of viral positive samples and total numbers of the tested engorged mosquitoes, respectively. The results present cumulative numbers from three independent experiments (Table S4). Statistics were performed using Fisher's exact test. Asterisks (*) and (***) indicate significance at *P* < 0.05 and < 0.001, respectively; **ns** indicates no significant difference. (D) Viral genome copies of USUV and **E**, WNV in the infected mosquito bodies after either a single or co-infectious blood meal exposure. One-way ANOVA with Tukey's multiple comparison was used to compare the mean of the genome copies. Asterisk (*) indicates a significant difference (*P* < 0.05); **ns** indicates no significant difference. The cut-off value for USUV and WNV genomes copies was indicated by black dash lines.

To investigate the effect of co-infection on the vector competence for both viruses, *Cx. pipiens* mosquitoes were exposed to both viruses simultaneously via an infectious blood meal. Viral infection in the bodies (proxy for infection) and saliva (proxy for transmission) was determined 14 days after the blood meal (Figure 4(A)). The percent ages of the infected mosquito bodies/saliva of the total tested engorged mosquitoes represent the infection rates and transmission rates, respectively.

After a single USUV infectious blood meal, 50% (84/169) of the total engorged mosquitoes were found to be infected, and 15% (25/169) of them contained USUV in their saliva (Figure 4(B)). Bloodmeal containing both viruses (co-exposure), however, significantly lowered USUV infection (36%, 54/152) and transmission (3%, 4/152) rates (fisher's exact test, *p* < 0.05 and *p* < 0.001, respectively) (Figure 4(B)). In contrast, WNV infection and transmission rates were comparable between mosquitoes that had ingested blood meals containing either solely WNV or both viruses, although a minor reduction in WNV transmission rate was observed (Figure 4(C)). This indicates that WNV outcompetes USUV when mosquitoes are simultaneously exposed to both viruses. A closer look at the infection and transmission rates after the co-exposure shows that 40 out 152 of the blood-fed mosquitoes were positive for both viruses (supplementary Table S4). From all these 40 mosquitoes with detectable co-infection in the body homogenate, only one of the mosquito saliva samples was positive for both USUV and WNV.

Sufficient viral replication inside the mosquito vector is a prerequisite for effective arbovirus dissemination and transmission [35]. We therefore looked at the infected mosquitoes and asked if the limited USUV transmission rate after co-exposure is due to reduced viral replication in the bodies. The results showed that the mean USUV viral genome copies were lower in the co-infected mosquitoes compared to that of the single USUV infected mosquitoes with a marginal significance (one-way ANOVA, F (2, 50) = 2.769, *p* = 0.0724) (Figure 4(D)). Interestingly, WNV viral genome copies were also lower in the co-infected mosquitoes compared to that of the single infected ones (one-way ANOVA, F (2, 55) = 3.632, *p* = 0.0330) (Figure 4(E)). This indicates that replication of both viruses was compromised in the co-infected mosquitoes.

### *Culex pipiens* mosquito pre-infection with USUV via blood meal significantly reduced WNV infection and transmission rates

**Figure 5.**
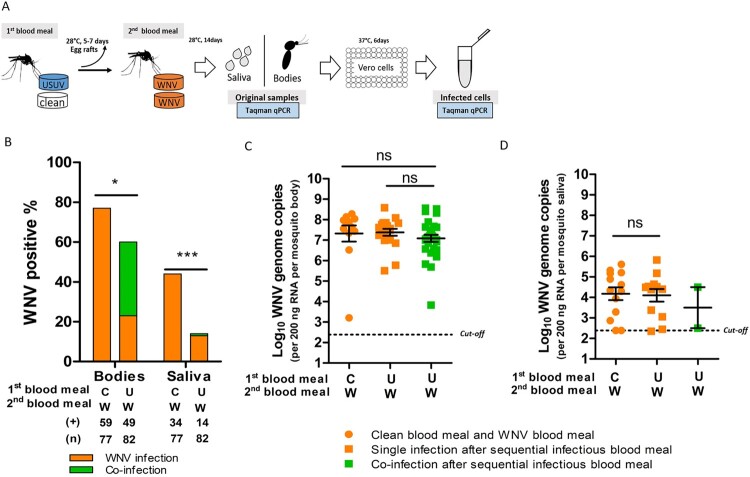
*Culex pipiens* pre-exposed to USUV infectious blood meal show a decreased WNV infection and transmission rate. (A) Schematic overview of the sequential blood meal experiment design. (B) Bar graph shows the percent of WNV positive mosquito bodies and saliva of the total engorged mosquitoes at 14 days after the WNV blood meal. “**C**”, “**U**” and “**W**” represent virus-free, USUV and WNV infectious blood meal, respectively. (**+**) and (**n**) indicate the numbers of WNV positive mosquito bodies/saliva and the total numbers of the engorged mosquitoes, respectively. The results present cumulative numbers from four independent experiments (Table S5). Fisher’s exact test was performed on the cumulative data. Asterisks (*) and (***) indicate significance at *P* < 0.05 and < 0.001, respectively. (C) WNV genome copies in mosquito bodies and (D) saliva after a sequential blood meal exposure. Kruskal-Wallis with Dunn's multiple comparison or *t* test was used to compare the mean of the genome copies among three or two data sets, respectively. **ns** indicates no significant difference. Black dash lines represent the cut-off value for WNV genome copies which corresponds to a Ct value of 34.

Because WNV outcompetes and reduces USUV infections both in cells and vector mosquitoes, we asked whether an established infection with USUV could affect the outcome of a subsequent infection with WNV in the vector mosquito. To study this, *Cx. pipiens* were pre-exposed to either an USUV infectious blood meal or virus-free blood meal before being challenged with a WNV infectious blood meal (Figure 5(A)). WNV infection rate and transmission rate were then determined at 14 days after the WNV blood meal.

The results show that pre-exposure to an USUV blood meal made *Cx. pipiens* less susceptible to subsequent WNV oral infection compared to mosquitoes without previous USUV exposure (77% (59/77) to 60% (49/82), fisher's exact test, *p* < 0.05) (Figure 5(B)). After the USUV and WNV sequential blood meals, only 17% (14/82) of the engorged mosquitoes contained WNV in their saliva, which is much lower compared to that of the mosquitoes exposed to the control, a virus-free blood meal, prior to oral infection with WNV (44% (34/77); fisher's exact test, *p* < 0.001) (Figure 5(B)). This indicates that pre-infection with USUV significantly inhibits the subsequent WNV infection and transmission rates. USUV and WNV sequential infectious blood meal lead to co-infections in 30/82 of the tested engorged mosquitoes, with an additional 19 USUV and 19 WNV single infection, respectively (Table S5). Interestingly, among the 30 co-infected mosquitoes, only 2 of them contained both USUV and WNV in their saliva. Together with the simultaneous infectious blood meal experiments, we only observed 3 mosquito saliva out of 70 co-infected mosquitoes that contain both viruses, indicating that the chance of concurrent transmission of both viruses via a single mosquito bite is low.

When looking at the WNV genome copies in the mosquito bodies, no significant reduction in the mean viral genome copies in the co-infected mosquitoes compared to the single infected ones can be observed (Figure 5(C)). A difference of WNV genome copies in the saliva between the co-infected and single infected mosquitoes cannot be determined due to only 2 co-infected saliva samples (Figure 5(D)).

### Involvement of the mosquito midgut in the USUV-mediated reduction of WNV infection and transmission

**Figure 6.**
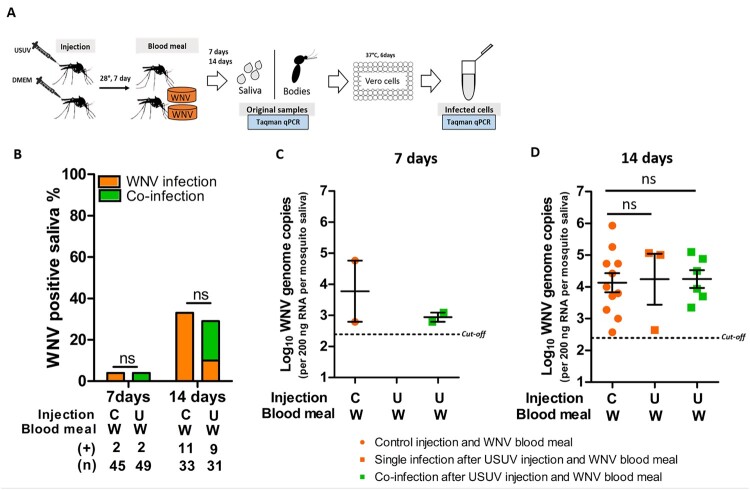
*Culex pipiens* pre-infection with USUV via injection did not inhibit subsequent WNV transmission rate. (A) Schematic overview of the sequential infection experiment design. (B) Bar graph shows the percent of WNV positive mosquito saliva of the total engorged mosquitoes at both 7 and 14 days after the WNV blood meal. “**C**” and “**U**” represent virus-free and USUV injection, respectively; “**W**” represents WNV infectious blood meal. **(+)** and **(n)** indicate the numbers of WNV positive mosquito saliva and the total numbers of the tested engorged mosquitoes, respectively. The results present cumulative numbers from three independent experiments (Table S6). WNV transmission rate was compared using Fisher's exact test. (C) WNV genome copies in mosquito saliva at 7 days and (D) 14 days after blood meal exposure. One-way ANOVA with Tukey's multiple comparison was used to compare the mean of the WNV genome copies among each groups. **ns** indicates no significant difference. Black dash lines represent the cut-off value for WNV genome copies.

We hypothesized that the observed reduction in WNV infection and transmission rates in mosquitoes pre-infected with USUV via blood meal is due to competition in the midgut cells. To investigate whether this is the case, USUV pre-infection was established by intrathoracic injection, which bypasses the mosquito midgut barrier. A WNV blood meal was offered 7 days after the injection and WNV transmission rate was determined at 7- and 14-days post the blood meal (Figure 6(A)). At the time of the WNV blood meal, USUV had already reached the saliva in most of the injected mosquitoes (Figure S4). Both 7 and 14 days after the WNV blood meal, no differences were observed between mosquitoes that received prior USUV injections or non-infectious control injections (containing DMEM) (Figure 6(B)). USUV pre-injection did not alter the level of WNV genome copies in the saliva compared to the control pre-injection with DMEM (Figure 6(C,D)). Therefore, the reduced WNV infection and transmission is likely due to competition with USUV in the mosquito midgut.

## Discussion

To date, little is known about the potential for USUV and WNV co-infections and how this can affect the transmission dynamics of both viruses. Here, we set up co-infection studies between contemporary isolates of USUV and WNV in cell cultures and vector mosquitoes. We observed a marked competitive advantage of WNV over USUV in three diverse cell types (mosquito, mammal and avian) during co-infection. It is not entirely clear how WNV outcompetes USUV in these cells, however, the relative higher speed of replication of WNV compared to USUV is likely the most logical explanation for this observation.

Our results showed for the first time, that *Cx. pipiens*, the primary vector species for both USUV and WNV, selectively transmits WNV when co-exposed to both viruses via an infectious blood meal (Figure 4). Previous laboratory studies have shown that the yellow fever mosquito *Aedes aegypti* can concurrently transmit the flaviviruses Zika virus (ZIKV) and dengue virus-2 (DENV-2), as well as the alphavirus chikungunya virus (CHIKV) after simultaneous exposure to these viruses via blood meal [5,6]. In contrast to our observation, authors from these two studies observed no significant interference in the infection rate or transmission rate between any of these viruses. Perhaps this can be explained by the evolutionary distance between these arboviruses. USUV and WNV are *Culex*-associated flaviviruses which are phylogenetically divergent from the *Aedes*-associated flaviviruses (e.g. ZIKV and DENV). USUV and WNV belong to the Japanese encephalitis virus (JEV) antigenic complex and as such share a relatively high similarity on both nucleotide (69%) and amino acid (76%) level, whereas ZIKV and DENV-2 are relatively more divergent (58% similarity on nucleotide and 56% on amino acid level), while CHIKV belongs to a completely different virus family [36]. Whether arboviruses with a high sequence homology are more prone to interfere with each other in the mosquito vector requires more investigations.

In regions where multiple arboviruses are co-circulating, successive blood meals may result in a sequential introduction of different pathogens into the mosquito. Since USUV has a larger geographic distribution than WNV in North-western European countries, an interesting question to ask is how the emerging WNV outbreak is affected by the presence of USUV-positive mosquitoes. Our results showed that mosquitoes orally exposed to USUV prior to WNV challenge are less competent for WNV transmission, indicating that pre-infection of USUV can “protect” the mosquitoes from WNV. Interestingly, when USUV was injected into the mosquitoes, these mosquitoes were not protected from subsequent infection with WNV, thus the mosquito midgut plays an important role in USUV-WNV competition. In a previous report, it has been shown that injection of an insect-specific virus (ISV) Palm Creek virus (PCV) to *Culex annulirostris* mosquito significantly inhibited WNV oral infection and transmission [37]. The authors also found that the injected PCV is specifically localized in the midgut epithelium cells. Therefore, they concluded that the WNV transmission inhibition may be caused by replication exclusion in the midgut cells. The ISV-induced exclusion of flaviviruses has also been observed in an *in vitro* study where mosquito cells previously infected with PCV were found less permissive to WNV and Murray Valley encephalitis virus (MVEV) [38]. Besides, the infection of ISV may cause the insertion of viral elements into the mosquito genome and these nonretroviral integrated RNA virus sequences (NIRVs) or endogenous viral elements (EVEs) are known to generate PIWI-Interacting RNAs which might regulate arbovirus replication [39,40]. Instead of competition, sequential exposure of two heterologous arboviruses, CHIKV and ZIKV to *Ae. aegypti* mosquitoes resulted in an transient enhancement of ZIKV transmission at 7 dpi but not at 12 dpi [41]. This suggests that the outcomes of the arbovirus-arbovirus or ISV-arbovirus co-infection and sequential infection may depend on the combination of viruses as well as the mosquito species.

Viral replication in the mosquito body, especially in the midgut, is essential for arboviruses to spread systemically and accumulate in the saliva. We observed a downregulation trend of both viral genome copies in the co-infected mosquito bodies compared to the single infected ones, which potentially contributes to the lower transmission rate of both viruses. We note that we investigated the presence of virus and viral RNA in mosquito bodies and saliva samples. How both viruses disseminate into specific tissues during co-infection remains a topic for future studies. For example, visualizing viral proteins in different tissues after co-infection may provide more details of where and when interference between USUV and WNV occurs inside the mosquitoes. At the cellular level, primary viral infection may induce the local immune response in the midgut cells which restricts subsequent viral infection and transmission, a phenomenon known as superinfection exclusion. This phenomenon is considered as a protection strategy for the primary virus to avoid competition from related secondary virus in a same host and has been observed in various flaviviruses and mosquito cells [42-45]. Given that antiviral immunity in mosquitoes is predominantly dictated by antiviral RNA interference (RNAi) [46], it could be hypothesized that USUV-derived small interfering (si)RNAs are able to block subsequent WNV infection and *vice versa*. However, when we conducted nucleotide sequence alignment of the complete USUV and WNV genomes, we only found three spots that show nucleotide homology of sequences longer than 21 nt in the beginning of the capsid coding region and the 3′ untranslated region. This suggests that USUV- or WNV- derived siRNAs are not very likely to silence WNV or USUV replication, respectively. To experimentally address the potential for RNAi, we also mapped the siRNA reads of USUV infected *Culex pipiens* mosquitoes on the WNV genome and *vice versa*, using the siRNA data sets generated from previous study [8], and found no significant matches, even when we allowed up to 1 mismatch between USUV siRNAs and the WNV genome (Figure S5). Thus, the competition in mosquitoes is most likely independent of a specific immune response, which is in line with the results obtained from cell lines.

The implication of arbovirus co-transmission on the epidemiology of these viruses remains unclear. With the average lifespan of female *Cx. pipiens* mosquitoes of approximately one month at 28°C [47], the competition we observed between USUV and WNV during our 3 weeks long sequential experiment suggests that USUV can play a significant and persistent role in reducing the ability of a mosquito to transmit WNV. This competitive relation between USUV and WNV may also impact the epidemiology of WNV in Europe. In regions where both viruses co-circulate, vector competence of the USUV infected mosquitoes for WNV might be reduced. In addition, the circulation of USUV in the WNV-free regions, may also impede WNV transmission and spread. Future field studies on the prevalence of both USUV and WNV in vector mosquitoes and host species across Europe are important to better understand the impact of USUV circulation on the geographic distribution of WNV.

## Supplementary Material

Supplementary_materials-revision_2.docx
