# Supplementary material for: Competition between Usutu virus and West Nile virus during simultaneous and sequential infection of Culex pipiens mosquitoes
Source: Emerg Microbes Infect. 2020 Dec 14;9(1):2642–52. doi: 10.1080/22221751.2020.1854623 (PMC7738303; doi:10.1080/22221751.2020.1854623)
Supplement: Supplementary_materials-revision_2.docx [file TEMI_A_1854623_SM7779.docx]

**Supplementary materials**

**Competition between Usutu virus and West Nile virus during simultaneous and sequential infection of *Culex pipiens* mosquitoes**

Haidong Wang^1^, Sandra R. Abbo^1^, Tessa M. Visser^2^, Marcel Westenberg^3^, Corinne Geertsema^1^, Jelke J. Fros^1^, Constantianus J. M. Koenraadt^2^, Gorben P. Pijlman^1*^

1. Laboratory of Virology, Wageningen University & Research, Wageningen, The Netherlands,

2. Laboratory of Entomology, Wageningen University & Research, Wageningen, The Netherlands,

3. Dutch National Plant Protection Organization (NPPO-NL), Wageningen, The Netherlands

*Corresponding to: gorben.pijlman@wur.nl

**Table S1 Primers and probes**

| **Primers/probe** | **Genome position** | **Sequence 5' to 3'** | **Modification** | **GenBank accession** |
| --- | --- | --- | --- | --- |
| USUVqF4 | NS5, 8955 | GGCTGTAGAGGACCCTCGG | none | MH891847.1 |
| USUVqR4 | NS5, 9098 | GACTGCCTTTCGCTTTGCCA | none |  |
| USUV probe | NS5, 9002 | TGTGTGGCACTCTCCTTTCAGATGGTTCT | 5’TexRd, 3’BHQ1 |  |
| T7 USUV F | NS5, 8677 | **TAATACGACTCACTATAGGG**AGCCCTGGGATGCAATTCTC | none |  |
| USUV R | NS5, 9589 | GTTCCTGCCCAATCACTCC | none |  |
| WNVqF4 | NS5, 8946 | AAGAACGCCCGGGAAGCC | none | HQ537483.1 |
| WNVqR4 | NS5, 9103 | TGCTGCCTTTAGCTTTGCCG | none |  |
| WNV probe | NS5, 9034 | TGTTGCATTCTCCACGGAGATGCGC | 5’FAM, 3’BHQ3 |  |
| T7 WNV F | NS5, 8617 | **TAATACGACTCACTATAGGG**GAAGTGAAACCAACCGGCTCAG | none |  |
| WNV R | NS5, 9526 | AGGTGTTCAGGGCGTAAGTC | none |  |

**Table S2 Specificity of the TaqMan qPCR system**

| **Dilution of USUV RNA** | | **Ct** | |
| --- | --- | --- | --- |
| **ng/reaction** | **genome copies/reaction** | **USUV** | **WNV** |
| 3.48E+01 | 7.14E+10 | 3.54 | 29.09* |
| 3.48E+00 | 7.14E+09 | 7.64 | 32.39* |
| 3.48E-01 | 7.14E+08 | 11.74 | 34.32 |
| 3.48E-02 | 7.14E+07 | 15.27 | 35.63 |
| 3.48E-03 | 7.14E+06 | 18.91 | 35.46 |
| 3.48E-04 | 7.14E+05 | 22.19 | Not detected |
| H2O |  | 36.38 | Not detected |
| **Dilution of WNV RNA** | | **Ct** | |
| **ng/reaction** | **genome copies/reaction** | **USUV** | **WNV** |
| 3.60E+01 | 7.39E+10 | 36.45 | 3.65 |
| 3.60E+00 | 7.39E+09 | 34.23 | 7.43 |
| 3.60E-01 | 7.39E+08 | 35.24 | 10.81 |
| 3.60E-02 | 7.39E+07 | 34.88 | 14.40 |
| 3.60E-03 | 7.39E+06 | 34.43 | 17.82 |
| 3.60E-04 | 7.39E+05 | Not detected | 21.17 |
| H2O |  | Not detected | 39 |

*When USUV RNA was in a very high amount (Ct < 10) and WNV is absent, WNV Ct is sometimes also around 30, indicating a minor cross-reactivity between WNV probe and high USUV RNA.

**Table S3. ANOVA test shows USUV experiences competition from WNV**

| **Viral genome copies** | **Factors** | **Df** | **Sum.Sq** | **Mean.Sq** | **F.value** | **Pr..F.** |
| --- | --- | --- | --- | --- | --- | --- |
| WNV | MOI_USUV | 1 | 1.57 | 1.57 | 0.15 | 7.04E-01 |
| WNV | MOI_WNV | 1 | 289.77 | 289.77 | 27.10 | **5.77E-06*** |
| WNV | Cells | 3 | 7.62 | 2.54 | 0.24 | 8.70E-01 |
| WNV | MOI_USUV:MOI_WNV | 1 | 0.52 | 0.52 | 0.05 | 8.26E-01 |
| WNV | Residuals | 41 | 438.37 | 10.69 |  |  |
|  |  |  |  |  |  |  |
| USUV | MOI_USUV | 1 | 69.21 | 69.21 | 58.48 | **2.04E-09*** |
| USUV | MOI_WNV | 1 | 49.12 | 49.12 | 41.50 | **1.02E-07*** |
| USUV | Cells | 3 | 27.69 | 9.23 | 7.80 | 3.12E-04* |
| USUV | MOI_USUV:MOI_WNV | 1 | 35.64 | 35.64 | 30.11 | **2.31E-06*** |
| USUV | Residuals | 41 | 48.52 | 1.18 |  |  |

***Term significant at alpha = 0.05**

To explore the relation between viral genome copies and MOI combination during co-infection, an ANOVA model (Viral genome copies = MOI_USUV + MOI_WNV + cell + MOI_USUV * MOI_WNV) was performed using R (v3.5.3, x64). The test result shows that WNV genome copies was only significantly related to WNV MOI (ANOVA, F = 27.10, df = 1, p < 1.00E-05); whereas USUV genome copies was significantly related to USUV MOI (ANOVA, F = 58.48, df = 1, p < 1.00E-08), WNV MOI (ANOVA, F = 41.50, df = 1, p < 1.00E-06) and the interaction between USUV and WNV MOI (ANOVA, F = 30.11, df = 1, P < 1.00E-05). This indicated that USUV experiences clear competition from WNV during co-infection.

**Table S4. Experiments of simultaneous infectious blood meal**

| **Experiments** | **Infectious blood meal** | ***n*^a^** | **Positive bodies** | | | **Infection rate**  **(Positive bodies/*n*)** | | **Positive saliva** | | | **Transmission rate**  **(Positive saliva/*n*)** | |
| --- | --- | --- | --- | --- | --- | --- | --- | --- | --- | --- | --- | --- |
|  |  |  | **USUV only** | **WNV only** | **Co-infection** | **USUV** | **WNV** | **USUV only** | **WNV only** | **Co-infection** | **USUV** | **WNV** |
| 1 | USUV | 52 | 36 | / | / | / | / | 11 | / | / | / | / |
| 2 |  | 55 | 31 | / | / | / | / | 9 | / | / | / | / |
| 3 |  | 62 | 17 | / | / | / | / | 5 | / | / | / | / |
| **Total** |  | **169** | **84** | / | / | **50% (84/169)** | / | **25** | / | / | **15% (25/169)** | / |
| 1 | WNV | 38 | / | 21 | / | / | / | / | 14 | / | / | / |
| 2 |  | 41 | / | 24 | / | / | / | / | 17 | / | / | / |
| 3 |  | 66 | / | 15 | / | / | / | / | 5 | / | / | / |
| **Total** |  | **145** | / | **60** | / | / | **41% (60/145)** | / | **36** | / | / | **25% (36/145)** |
| 1 | USUV+WNV | 37 | 5 | 6 | 6 | / | / | 0 | 8 | 0 | / | / |
| 2 |  | 52 | 8 | 8 | 17 | / | / | 3 | 14 | 1 | / | / |
| 3 |  | 63 | 1 | 9 | 17 | / | / | 0 | 4 | 0 | / | / |
| **Total** |  | **152** | **14** | **23** | **40** | **36% (54/152)** | **41% (63/152)** | **3** | **27** | **1** | **3% (4/152)** | **18% (27/152)** |

^a^ Sample size (*n*) shown are the numbers of fully engorged mosquitoes for salivation assay at 14 days post the oral infection

**Table S5. Experiments of sequential infectious blood meal**

| **Experiments** | **1st blood meal** | **2nd blood meal** | ***n*^a^** | **Positive bodies** | | | **WNV Infection rate**  **(positive bodies/*n*)** | **Positive saliva** | | | **WNV transmission rate**  **(positive saliva/*n*)** |
| --- | --- | --- | --- | --- | --- | --- | --- | --- | --- | --- | --- |
|  |  |  |  | **USUV only** | **WNV only** | **Co-infection** |  | **USUV only** | **WNV only** | **Co-infection** |  |
| 1 | Virus-free | WNV | 14 | / | 11 | / | / | / | 8 | / | / |
| 2 |  |  | 24 | / | 18 | / | / | / | 5 | / | / |
| 3 |  |  | 19 | / | 15 | / | / | / | 11 | / | / |
| 4 |  |  | 20 | / | 15 | / | / | / | 10 | / | / |
| **Total** |  |  | **77** | **/** | **59** | **/** | **77% (59/77)** | **/** | **34** | **/** | **44% (34/77)** |
| 1 | USUV | WNV | 10 | 0 | 8 | 1 | **/** | 0 | 4 | 0 | / |
| 2 |  |  | 18 | 1 | 8 | 3 | **/** | 3 | 5 | 0 | / |
| 3 |  |  | 27 | 6 | 1 | 17 | **/** | 19 | 2 | 0 | / |
| 4 |  |  | 27 | 12 | 2 | 9 | **/** | 17 | 1 | 2 | / |
| **Total** |  |  | **82** | **19** | **19** | **30** | **60% (49/82)** | **39** | **12** | **2** | **17% (14/82)** |

^a^ Sample size (*n*) shown are the numbers of fully engorged mosquitoes for salivation at 14 days post the 2^nd^ blood meal exposure.

**Table S6 Replicate experiments of sequential injection and infectious blood meal**

| **Experiments** | **Injection** | **Blood meal** | ***n***^a^ **(7dpi)** | **WNV positive**  **saliva (7dpi)** | | | **WNV transmission rate 7dpi**  **(positive saliva/*n*)** | ***n***^a^ **(14dpi)** | **WNV positive**  **saliva (14 dpi)** | | | **WNV transmission rate 14 dpi**  **(positive saliva/*n*)** |
| --- | --- | --- | --- | --- | --- | --- | --- | --- | --- | --- | --- | --- |
|  |  |  |  | **USUV only** | **WNV only** | **Co-infection** |  |  | **USUV only** | **WNV only** | **Co-infection** |  |
| 1 | DMEM | WNV | / | / | / | / | / | 14 | 0 | 4 | 0 | / |
| 2 |  |  | 20 | 0 | 2 | 0 | / | 19 | 0 | 7 | 0 | / |
| 3 |  |  | 25 | 0 | 0 | 0 | / | / | / | / | / | / |
| **Total** |  |  | **45** | **0** | **2** | **0** | **2/45** | **33** | **0** | **11** | **0** | **11/33** |
| 1 | USUV | WNV | / | / | / | / | / | 7 | 4 | 0 | 1 | / |
| 2 |  |  | 21 | 18 | 0 | 2 | / | 24 | 10 | 3 | 5 | / |
| 3 |  |  | 28 | 26 | 0 | 0 | / | / | / | / | / | / |
| **Total** |  |  | **49** | **44** | **0** | **2** | **2/49** | **31** | **14** | **3** | **6** | **9/31** |

^a^ Sample size (*n*) shown are the numbers of fully engorged mosquitoes for salivation at 7 days and 14 days post the blood meal.


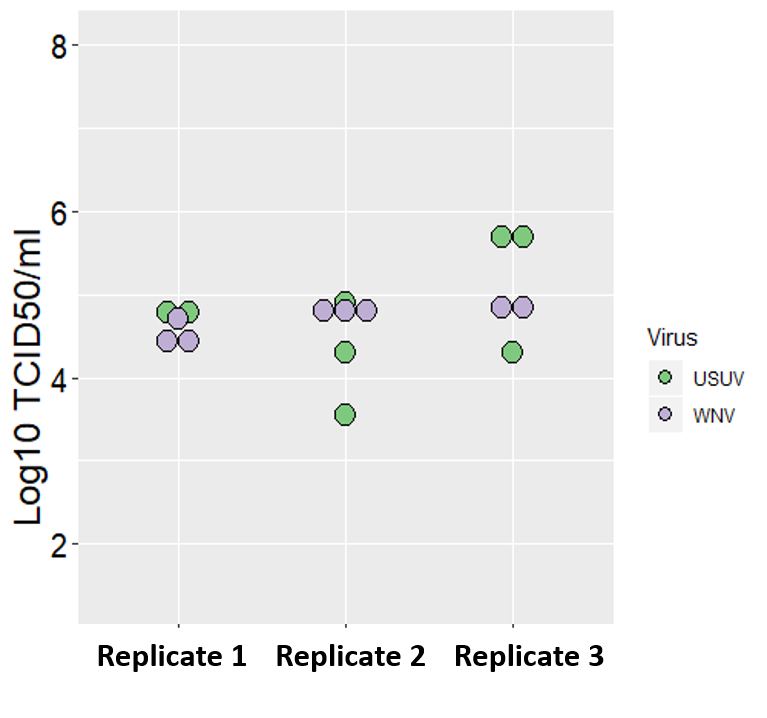


**Figure S1. USUV and WNV titers in the engorged mosquitoes after blood meal from three independent replicate experiments.** After each blood meal, 3 engorged mosquitoes were stored in -80°C to check the virus titers. Shown are USUV and WNV virus titers in mosquitoes after either USUV or WNV single blood meal from three independent replicate experiments.


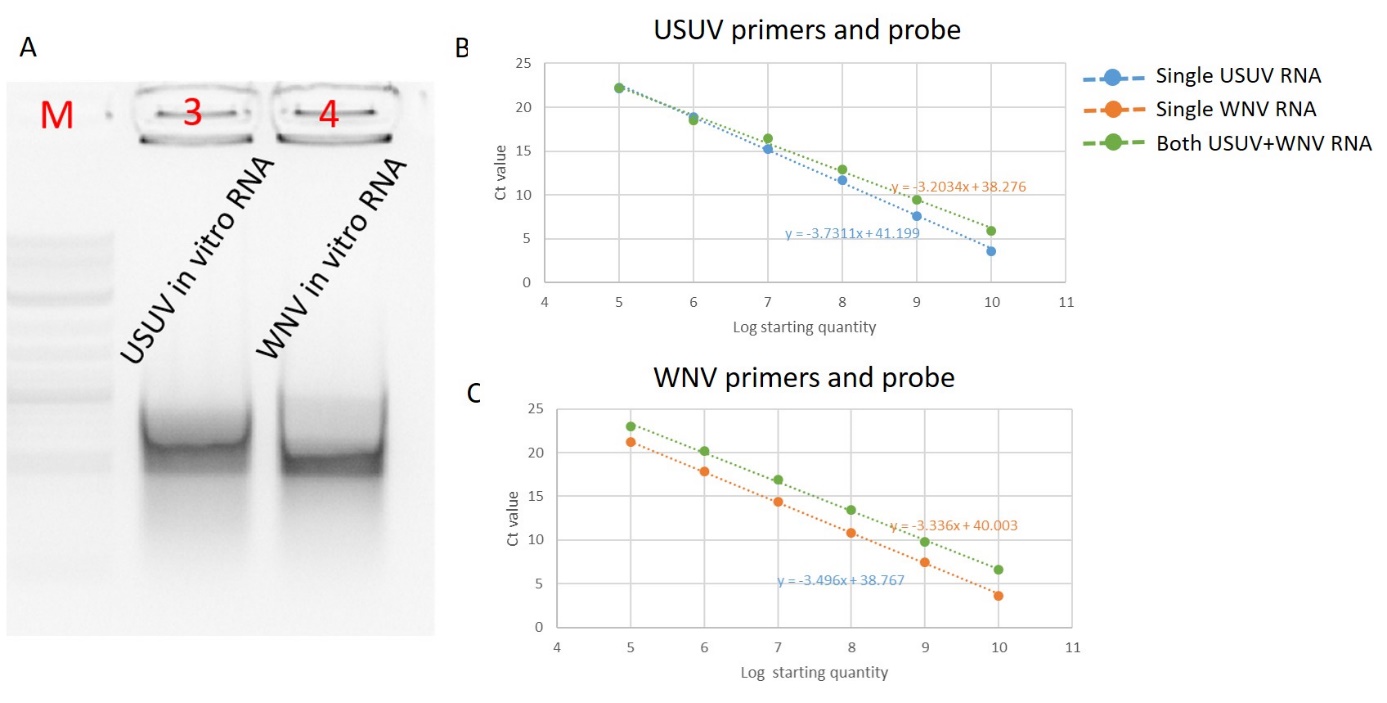


**Figure S2. Amplification efficiency (AE) of USUV and WNV Taqman qPCR probe.** A, *In vitro* T7 RNA of USUV and WNV. Amplification efficiency of USUV (B) and WNV (C) primers and probes on single RNA (USUV or WNV) and both RNA. Blue dots and dash lines indicate the presence of single USUV RNA, orange dots and dash lines indicate the presence of single WNV RNA, green dots and dash lines indicate the presence of both USUV and WNV RNA.


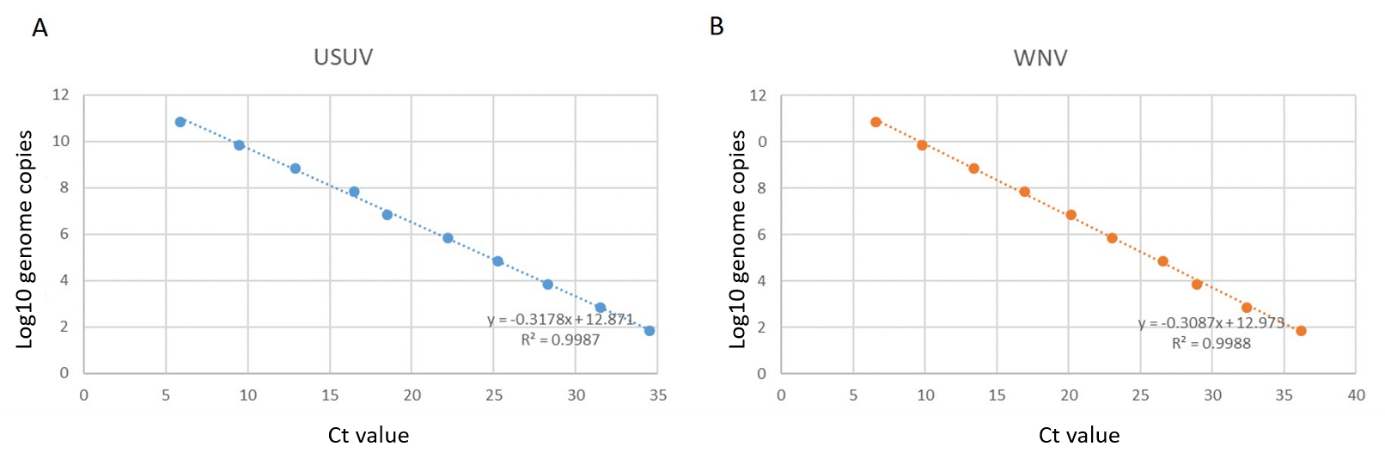


**Figure S3. Standard curve for USUV and WNV TaqMan primers and probe.** The *in vitro* transcript viral RNA was quantified by Nanodrop and used to make a 10-time dilution series. Viral genome copies were calculated by online tool (http://endmemo.com/bio/dnacopynum.php


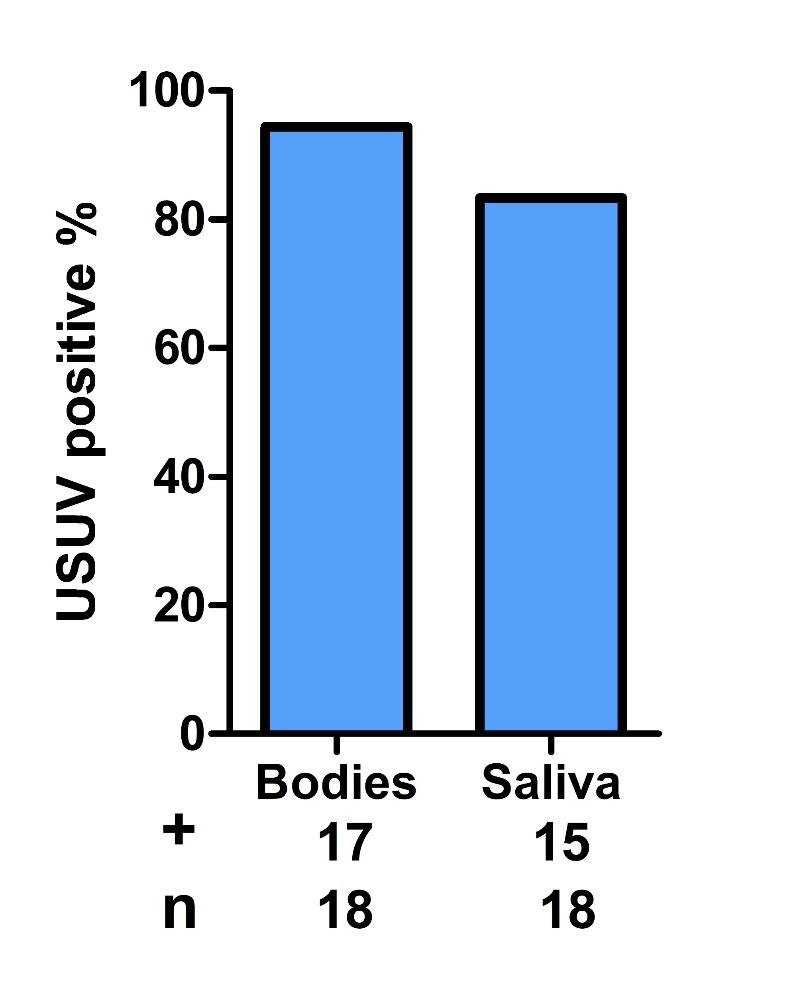


**Figure S4. USUV positive bodies and saliva 7 days after intrathoracic injection.** 69nl USUV P6 stock (approximately 2400 TCID50/ml) was injected intrathoracically with glass capillaries. After injection, the mosquitoes were kept for 7 days. Before WNV infectious bloodmeal, 6-8 mosquitoes from each experiment repeat were sacrificed to check the infection (body percent %) and transmission (saliva percent %) of USUV.


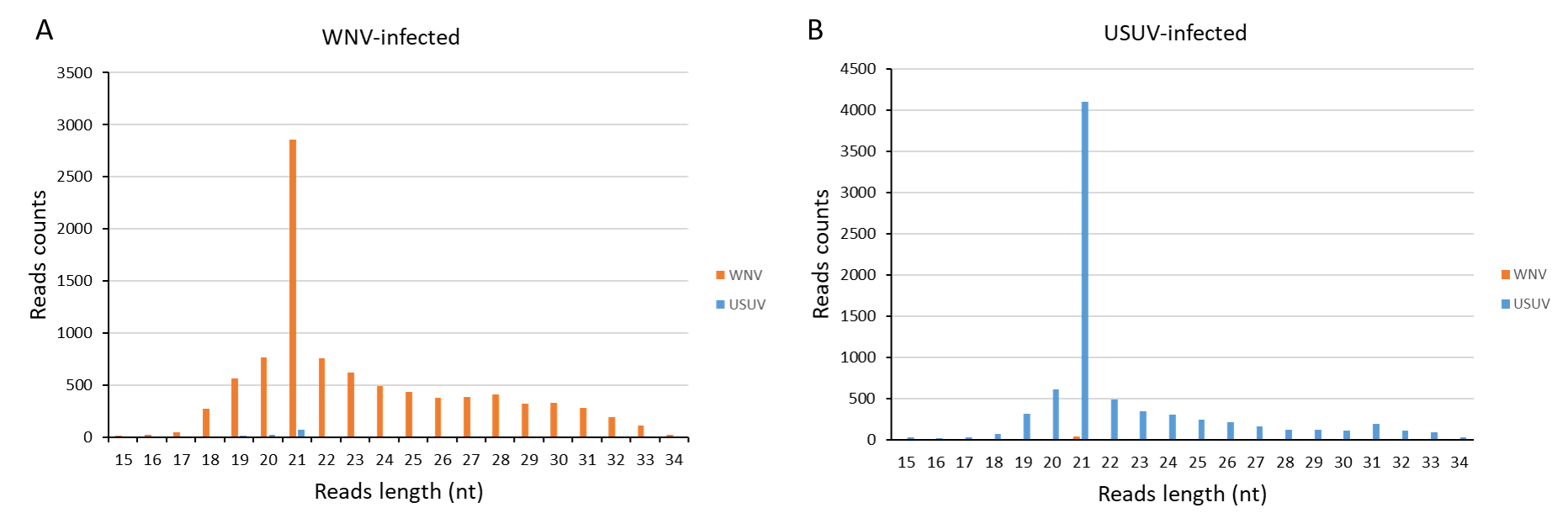


**Figure S5. Small interfering RNA (siRNA) data of USUV or WNV infected *Culex pipiens* mosquitoes. A,** siRNA reads derived from WNV-infected Culex pipiens mosquitoes were mapped to both WNV and USUV genome. **B,** siRNA derived from USUV-infected *Culex pipiens* mosquito were mapped to both USUV and WNV genome. Orange bars indicate the read counts for WNV, and blue bars represent the reads counts for USUV. The siRNA data was produced in the previous study [1].

1. Fros JJ, Miesen P, Vogels CB, et al. Comparative Usutu and West Nile virus transmission potential by local Culex pipiens mosquitoes in north-western Europe. One Health. 2015 Dec;1:31-36.
